# Three-Dimensional Localization of Buried Polyethylene Pipes Using Acoustic Method

**DOI:** 10.3390/s22239433

**Published:** 2022-12-02

**Authors:** William Xerri, Gineth Saracco, Alessandra Ribodetti, Laurent Zomero, Philippe Picon

**Affiliations:** 1 CNRS-UMR7330 CEREGE, AMU, CdF, IRD, INRAE, OSU-Pytheas, Europole de l’Arbois, BP 80, 13545 Aix-en-Provence, CEDEX 4, France; 2 MADE-SA, 167 Impasse de la Garrigue, 83210 La Farlède, France; 3 IRD-UR082 CNRS-UMR7329 GEOAZUR, Campus Azur Université et Observatoire de la Côte d’Azur, 250 rue Albert Einstein CS 10269, 06905 Sophia Antipolis, France

**Keywords:** acoustic method, buried polyethylene pipe, MUSIC algorithm, propagation time modeling, signal processing, full wavefield

## Abstract

Localization of buried polyethylene pipes is an important issue for network managers. This study focuses on an acoustic method, which consists of vibrating the pipe and observing the signal with a receiver placed on the ground surface. This method provides an estimate of the path of the pipe but gives no information on the depth. We developed a multi-sensor method based on the principle of vibrating the pipe, which allows estimating the depth while being non-invasive and non-destructive and without a priori information on the propagation medium. These sensors are positioned perpendicular to the pipe. We developed a new estimator to estimate the depth and the propagation velocity in the medium, which is an important variable in our problem. This estimator is based on the MUSIC algorithm and is adapted to our choice of modeling. In this paper, two models of travel times in typical situations are presented. The first one represents the case where all sensors can be placed inside the trench (on the ground surface) in which the pipe is buried. The second one represents the case where sensors are placed inside and outside the trench. These travel time models aim to provide a fast result to allow the method to be used by field agents. They are compared with a full wavefield modeling by finite differences.

## 1. Introduction

Different methods exist for locating buried pipes [1] and, more generally, for investigating near-surface structures [2,3,4,5,6,7]. The choice of method to locate buried pipes depends on the context. The use of tracer wires is becoming increasingly widespread. This method consists of burying an electric wire with the pipe, which allows, by passing a current through the wire, the creation of an electromagnetic field and, therefore, the use of electromagnetic methods. Electromagnetic methods provide very good results for locating pipes when they are applicable. If there is no tracer wire, then two possible cases can be discerned. Either the pipe is metallic, or it is not. In the case of a metal pipe, an electric current can be injected into the pipe; electromagnetic methods, which work very well, can, therefore, be used. In the case of non-metallic pipes, two types of processes can be distinguished in a non-exhaustive way: (i) Ground-Penetrating Radar (GPR) [8,9] is a versatile tool for locating buried infrastructures; however, this type of tool often needs to be calibrated according to the type of soil to which it is applied. Moreover, it is difficult to differentiate a water pipe from a gas pipe or a buried electrical cable. (ii) Acoustic methods are applicable in the case of non-conductive pipes [1,10,11,12]. According to [1], we can refer to several types of acoustic methods: seismic wave methods [13], point vibration measurements [14] and pipe excitation methods [15]. The first two categories can use a specific source placed on the ground surface for probing. These methods do not differentiate between the pipe of interest and other pipes present. What distinguishes the last category is its ability to vibrate the pipe, and this vibration will then be diffused into the ground.

This study focuses on the pipe excitation methods that have the advantage of discriminating the pipe of interest in a dense urban environment with noise.

The principle of this is to inject an acoustic signal (called acoustic signature) into the pipe. A method that has been used for many years in the field uses a receiver (geophone) placed on the ground surface, which measures the vertical vibrations of the ground. Through successive measurements of the energy of the received signal, this method can estimate the passage of the pipe and follow its path. However, this method does not provide any information on the pipe depth. The GasTracker tool, based on this method, was developed by the company MADE-SA. In this study, a GasTracker is used to estimate, experimentally on the ground surface, the X and Y position (at ±10 cm) of the pipe in order to position the sensor network in the area where the pipe is located.

The objective of this study is to implement a multi-sensor method, based on the differences in travel times between sensors, to estimate the depth in addition to the passage of the pipe [10,11].

The aim of this work is to estimate the depth with an accuracy below 10 cm with a non-destructive and non-invasive method and without any a priori information on the propagation medium (e.g., characteristics of the soil, which can change completely from one application area to another).

Nevertheless, some information on how the pipe is buried is known. The pipe is buried between 0.4 and 1.5 m. It is buried in a trench whose width can vary from approximately 30 cm to over 1 m. Therefore, two different media can be distinguished, the inside of the trench and the outside of the trench (vertical stratifications). Moreover, in this problem, the depth is at the meter scale.

An important aim of this study is to define a model of the travel time of the signal that provides the fastest result in order to directly estimate the depth in the field. In the travel time models presented in this paper, we consider the first arrival time of the signals. Only P-waves and converted S-waves are considered. Geophones used are single component sensors. They measure the vertical component of the received vibrations.

The travel time models that we propose are confronted using a simulation with much more complete propagation modeling than that of the complete wavefield with finite differences. This comparison aims to verify the coherence of the estimated quantities, in particular, the velocity.

A cylindrical scattering of the vibration induced by the pipe is considered [16,17,18,19]. To ensure that all sensors observe the same section of the vibrating pipe, they are placed perpendicular to the pipe. This is why the problem is represented in a plane orthogonal to the passage of the pipe. This section can be approximated by a point source.

The variables to be estimated in this problem are the position of this source, its lateral position S_X_ and especially its depth Sz, but also the average velocity of propagation in the ground. An average velocity is assigned to each medium of propagation.

In the first part, the sensors are assumed to be all positioned inside the trench. Therefore, a modeling of the travel times of the signal between the pipe and the receivers by considering a weakly heterogeneous medium and by assigning a mean propagation velocity is presented. In order to ensure an acceptable estimation accuracy, an estimator based on the MUSIC (Multiple Signal Classification) algorithm adapted to this modeling of travel times is developed. This estimator allows us to evaluate variables of interest (position of the source and the velocity). Measurements are carried out on a test area in order to work on real data. In the second part, the sensors are positioned inside and outside the trench. As an extension of the method developed in the first part, the modeling is adapted to this case to fit a larger number of situations. The different compaction of the soil on either side of the trench is taken into account. This requires considering a velocity specific to each of these environments. This second modeling of travel times is validated within the framework of the problem, initially through numerical simulations, then through comparison with finite differences, and finally by comparison with real data.

## 2. Modeling with a Single Propagation Medium (M1)

### 2.1. Propagation Time Modeling M1

**Figure 1:**
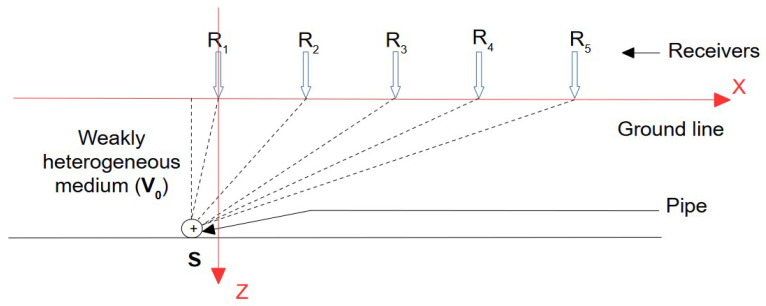
Scheme of Model M1: case of a single propagation medium.

In this section, all sensors are considered to be located inside the trench. Since no information on the characteristics of the propagation medium is known, the first approach is to consider the propagation medium as weakly heterogeneous and to assign an average propagation velocity. The problem is represented in a plane orthogonal to the passage of the pipe (Figure 1). The pipe section has been represented using a point source S, the receivers R_i_ were placed on the ground surface perpendicular to the pipe. An average velocity V_0_ has been assigned to the propagation medium. The position of the source S (S_X_; S_Z_) and the velocity of the wave V_0_ were unknown. The only known parameters were the coordinates of the receivers R_i_ (R_iX_; R_iZ_ = 0).

We defined $\theta_{M1}$ as the vector of variables to be estimated in the case of Model M1.

(1) $$\theta_{M1}\;=\;{\left[\begin{array}{ccc} S_{X} & S_{Z} & V_{0} \end{array}\right]}^{T}$$

Here, the symbol [.]^T^ is the transposed operator, S_x_ the plumb of the pipe, S_z_ the pipe depth and V_0_ is the average propagation velocity in the medium.

We note $\tau_{i}\left(\theta_{M1}\right)$ as the wave travel time between the source S and the receiver R_i_ for Model M1.

(2) $$\tau_{i}\left(\theta_{M1}\right)\;=\;\frac{\left|{\mathrm{SR}}_{i}\right|}{V_{0}}$$

We note $\tau_{1i}\left(\theta_{M1}\right)$ as the relative delay time between sensors R_1_ and R_i_ for Model M1.

(3) $$\tau_{1i}\left(\theta_{M1}\right)\;=\;\frac{\left|{\mathrm{SR}}_{i}|\;-\;|{\mathrm{SR}}_{1}\right|}{V_{0}}$$

Relative delay times are considered because, in this problem, the emission time of the source S is unknown, but the receivers are triggered synchronously.

### 2.2. Validation of Travel Time Estimate of M1 through Comparison with a Finite Difference Modeling of the Full Wavefield

In this section, the P-wave first arrival travel time estimation proposed in this paper is validated through comparison with full wavefield modeling using finite differences.

The acoustic and isotropic version of the code developed by Operto et al. is used [19] from the original formulation proposed by Jean Virieux [20,21].

A comparison of the travel time estimate of M1 through the finite difference modeling of the full P-wavefield is presented here, which is computationally fast and accurate in the presence of flat topography, as in the test site analyzed in this study.

The following case study has been performed: 30 sensors were aligned and spaced 0.01 m apart; sensor 1 (R_1_) was placed just above the source (S_X_ = 0); the rest of the sensors were placed increasingly far away from the source; the depth of the source (S_Z_) was 1.5 m, and the average velocity of propagation (V_0_) was 500 m/s.

**Figure 2:**
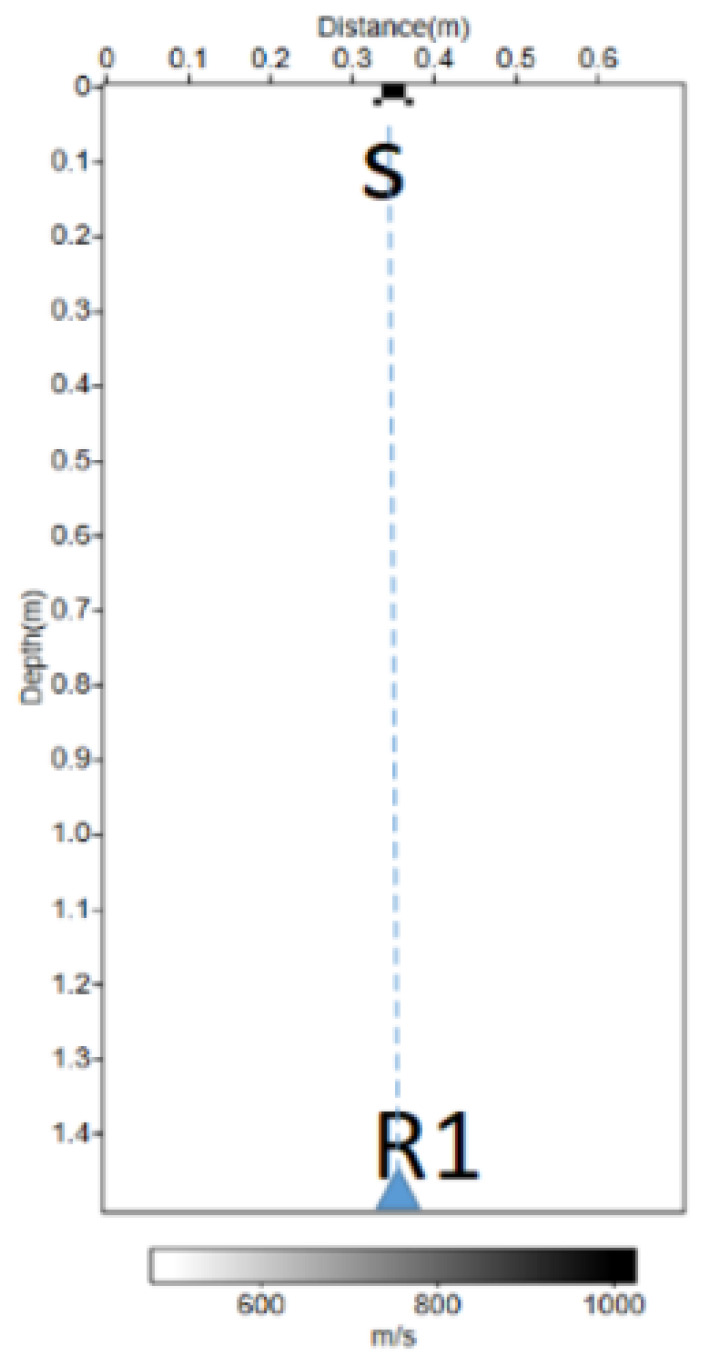
Physical velocity model in the case of the propagation of the full wavefield by finite differences with one propagation medium. S indicates the source at the surface. R_1_ is the first receiver.

To ensure simplicity in the simulation, the origin of the reference frame was translated, and the source was placed at the top and the sensors at the bottom, but they were symmetrical. In the simulation presented here, the source was placed at S (0.35; 0), and the sensors at Ri (0.35 + (i − 1) 0.01; 1.5), but this corresponds well to the situation described above. In Figure 2, we observe the physical model used for the simulation. The source used was a Ricker function, also called the “mexican hat”; this function is a second derivative of a Gaussian function.

**Figure 3:**
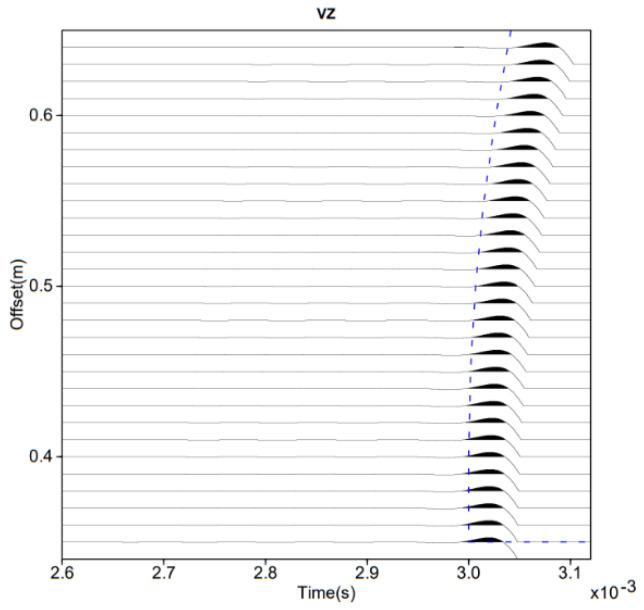
Comparison of travel time estimate of Model M1 (dashed blue line) with the full P-wavefield propagation model using finite differences.

The results of this simulation are presented in Figure 3. The vertical axis represents the spatial position of the sensors, and the horizontal axis shows the recorded times. The simulation of the received signals using finite differences is shown in black, and the travel time curve calculated using Model M1 is shown in blue. At the scale of the problem, Model M1 is in agreement with the more complex full wavefield model using finite differences.

### 2.3. Cramer–Rao Bound from Model M1

The Cramer–Rao Bound (CRB) was calculated to obtain information on the estimation accuracy [22,23]. The CRB represents the smallest possible standard deviations of all unbiased estimates of the model variables. First, the CRB was computed, and second, simulations using the CRB were performed.

#### 2.3.1. Calculation of the Cramer–Rao Bound from Model M1

The CRB was calculated according to the Fisher information matrix, which is denoted by F.

(4) $$\mathrm{CRB}\left(\theta_{M1}\right)\;=\;F^{-1}\left(\theta_{M1}/\tau_{1i}\left(\theta_{M1}\right)\right)$$

where ./. represents the known operator.

The Fisher information matrix is expressed

(5) $$F\left(\theta_{M1}/\tau_{1i}\left(\theta_{M1}\right)\right)\;=\;\overset{N}{\underset{i\;=\;2}{\sum }}\frac{1}{\mathrm{Var}\left(\tau_{1i}\left(\theta_{M1}\right)\right)}\nabla_{\theta_{M1}}\left(\tau_{1i}\left(\theta_{M1}\right)\right)\nabla_{\theta_{M1}}^{T}\left(\tau_{1i}\left(\theta_{M1}\right)\right)$$

where N is the number of sensors, $\mathrm{Var}\left(\tau_{1i}\left(\theta_{M1}\right)\right)$ is the variance of the relative delay time between sensor 1 and i, and $\nabla_{\theta_{M1}}\left[.\right]$ is the gradient operator as a function of $\theta_{M1}$.

Let us focus on the calculation of the gradient of $\tau_{1i}\left(\theta_{M1}\right)$. The expression of this gradient can be decomposed from Equation (3),

(6) $$\nabla_{\theta_{M1}}\left(\tau_{1i}\left(\theta_{M1}\right)\right)\;=\;\nabla_{\theta_{M1}}\left(\frac{\left|{\mathrm{SR}}_{i}\right|}{V_{0}}\right)\;-\;\nabla_{\theta_{M1}}\left(\frac{\left|{\mathrm{SR}}_{1}\right|}{V_{0}}\right)$$

By developing the calculation, the expression becomes

(7) $$\nabla_{\theta_{M1}}\left(\tau_{1i}\left(\theta_{M1}\right)\right)\;=\;\left[\;\begin{array}{c} \frac{S_{X}\;-\;R_{\mathrm{iX}}}{V_{0}|{\mathrm{SR}}_{i}|}\;-\;\frac{S_{X}}{V_{0}|{\mathrm{SR}}_{1}|}\\ \frac{S_{Z}\;-\;R_{\mathrm{iZ}}}{V_{0}|{\mathrm{SR}}_{i}|}\;-\;\frac{S_{Z}}{V_{0}|{\mathrm{SR}}_{1}|}\\ \frac{\left|{\mathrm{SR}}_{1}|\;-\;|{\mathrm{SR}}_{i}\right|}{V_{0}^{2}} \end{array}\;\right]$$

Using this result in Equation (5), the Fisher information matrix can be calculated, and by calculating its inverse, the CRB is obtained, as shown in Equation (4).

#### 2.3.2. Numerical Simulation Using the Cramer–Rao Bound

The figures presented in this section are the results of the numerical simulations. These results are not exhaustive and are used to give an idea of the impact of the variables of the problem on the accuracy of the depth estimation. To obtain these curves, one variable was varied and the others were fixed. The impact on the possible accuracy of depth estimation was observed.

**Figure 4:**
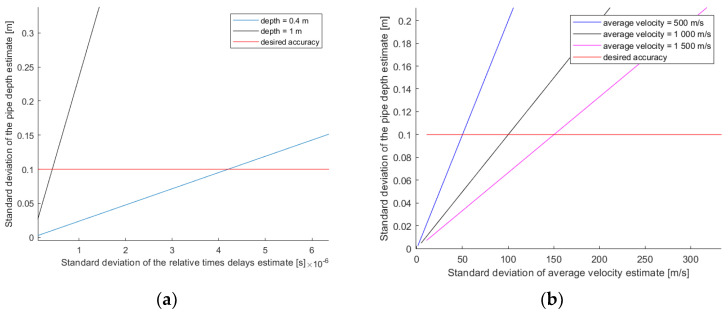
Depth error boundary as a function of the error of the other variables: (**a**) depth error as a function of the error in the relative delay times; (**b**) depth error as a function of the error in the propagation velocity.

Figure 4a shows the evolution of the CRB with changes in depth as a function of the error in the relative delay times. The situation considered for this simulation is as follows: five sensors were used, the distance between sensors was 0.2 m, the plumb of the pipe (S_X_) was placed at 0 m, and the average propagation velocity (V_0_) was 500 m/s. Curves for two different depths (S_Z_), 0.4 and 1 m, were observed. An accuracy of the relative delay times in a microsecond range was needed to obtain an accuracy of 0.1 m in the depth. In the worst case, an accuracy of the order of 0.1 μs was needed.

Figure 4b represents the evolution of the CRB with changes in depth as a function of the error in the propagation velocity. The situation considered for this simulation is as follows: the distance between the sensors was 0.2 m, the plumb of the pipe (S_X_) was 0 m, and the pipe depth (S_Z_) was 0.4 m. The curves for different average propagation velocities (V_0_) were observed. A propagation velocity accuracy of the 10% range was required to obtain an error of less than 0.1 m in the depth.

### 2.4. Adaptation of the MUSIC Algorithm for Model M1

In the previous section, a relative delay time accuracy of the order of 10^−6^ s was defined as a necessary condition. Following the choice to focus on the MUSIC algorithm (MUltiple SIgnal Classification) [24,25,26,27,28,29,30] for antenna processing, the so-called ‘high resolution algorithm’ became interesting.

#### 2.4.1. Presentation of the MUSIC Algorithm Adapted to Our Problem

The MUSIC algorithm is usually used to discern different sources and their direction of arrival through an antenna array [24]. It can be adapted in the near field to estimate the distance of sources in addition to their direction of arrival [25,26,27,28,29,30]. In this paper, the aim was to estimate the location of a single source S in the near field and also to estimate the propagation velocity V_0_ according to Model M1.

The algorithm can be divided into several steps as follow:

1. Estimate the variance–covariance matrix of the system from the signals received by sensors.
2. Decompose the variance–covariance matrix of the system into eigenvalues and eigenvectors.
3. Definition of the noise subspace, denoted by U_b_, with the eigenvectors corresponding to the smallest eigenvalues.
4. Construction of a family of vectors, denoted by ‘a’, parametrized by the variables we want to estimate, S and V0 (Equation (1)). This family of vectors is constructed from the modeling of relative delay times (Equation (3)).

(8) $$a\left(S,V_{0}\right)\;=\;{\left[\begin{array}{cccc} 1 & e^{-j2{\pi f}_{0}\frac{\left|{\mathrm{SR}}_{2}|\;-\;|{\mathrm{SR}}_{1}\right|}{V_{0}}} & \ldots & e^{-j2{\pi f}_{0}\frac{\left|{\mathrm{SR}}_{N}|\;-\;|{\mathrm{SR}}_{1}\right|}{V_{0}}} \end{array}\right]}^{T}$$

where f_0_ is the signal frequency, and N is the number of sensors.

5. Knowing that the signal subspace and the noise subspace are orthogonal, the project of a (S,V_0_) on the noise subspace must be at the minimum for the values of S and V_0_ corresponding best to the received signal. It is traditional to take the inverse of this projection and to look for the values of S and V_0_ that maximize this criterion, which is denoted by C_music_.

(9) $$C_{\mathrm{MUSIC}}\left(S,V_{0}\right)\;=\;\frac{1}{a{\left(S,V_{0}\right)}^{H}U_{b}{U_{b}}^{H}a\left(S,V_{0}\right)}$$

where [.]^H^ is the conjugate transposed operator.

#### 2.4.2. Test of Estimator Using Numerical Simulation

In this part, the algorithm used in the simulation of an ideal case is verified. The statistics of the estimator (mean and variance) using the Monte Carlo method are presented. All estimates were made with 1000 runs of the noise, and noise is applied to the propagation times. For these simulations, a situation has been fixed in which all the variables are known. The signals received are simulated as a function of these variables, and then these ones were used to run the algorithm.

The following situation is considered: a depth (S_Z_) of 0.7 m, a propagation velocity (V_0_) of 500 m/s, and the plumb of the pipe (S_X_) at 0 m. Five sensors were placed at 0.2 m, the first one vertically above the pipe.

In Table 1, the statistics of the results of the estimator, using the Monte Carlo method, are presented. A total of 1000 runs of noise were performed. The same situation as before was considered, and the theoretical propagation times between the source S and sensors were noised with a white Gaussian noise of standard deviation σ_noise_. The propagation times are in the millisecond range, and the propagation time differences with sensor 1 vary between 10^−5^ and 10^−4^ seconds.

For this set of variables, which is representative, the observed accuracy is lower than the desired accuracy of 0.1 m. It is interesting to note that increasing the number of sensors reduces the accuracy required on the relative delay times.

### 2.5. Experimental Measurements

Experimental measurements were performed on a semi-controlled test area in order to work on real data.

#### 2.5.1. Experimental Set-Up

The measurement chain presented in this section is not innovative; this measurement chain was designed to be adapted to experimental needs. In particular, it was necessary to be able to control the emission and to change the type of signal emitted at any time. Moreover, in the reception chain, the filter should not be so fine that it could not adapt to different types of signals that could be emitted.

**Figure 5:**
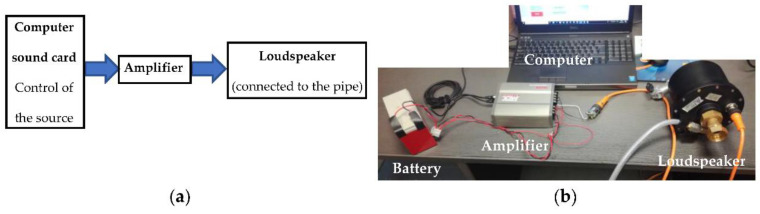
Experimental set-up of the acoustic emission chain: (**a**) diagram of the main components; (**b**) photograph of the material with the battery to power the amplifier.

The emission chain (Figure 5) was composed of a computer to control the emitted signal, an amplifier, which received the signal from the computer sound card, and finally, a loudspeaker. The loudspeaker was fixed at one end of the pipe and emitted the signal inside the pipe.

**Figure 6:**
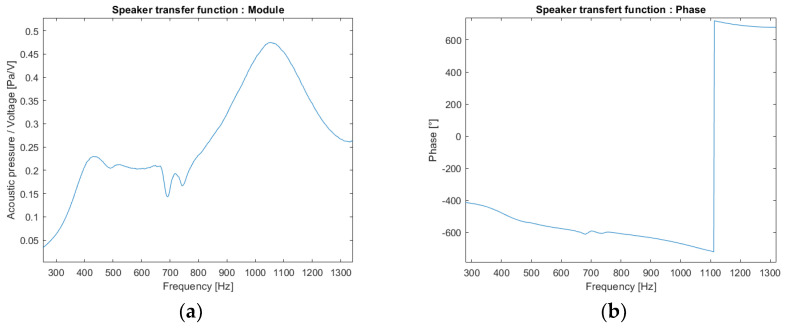
Transfer function of the loudspeaker estimated by emitting a white noise: (**a**) modulus; (**b**) phase.

Experiments were carried out in an anechoic room to characterize the loudspeaker (CNRS-LMA-Marseille, C. Pinhède). A white noise is sent in command to the loudspeaker, and the emitted signal is measured with a microphone placed just in front of the loudspeaker output. This experimentation allows to obtain the transfer function of the loudspeaker. The modulus and phase of its transfer function are shown in Figure 6.

The responses of the loudspeaker to the two types of signals used in this study are presented.

**Figure 7:**
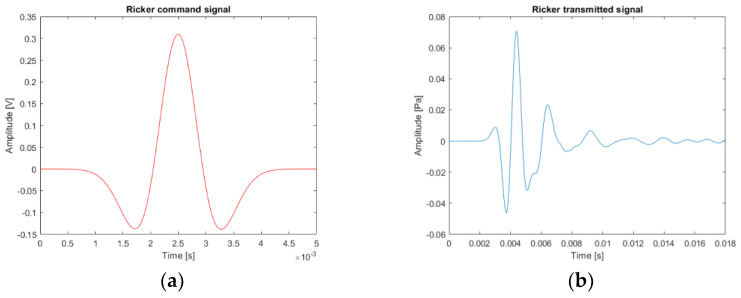
Example of the loudspeaker response (Ricker function): (**a**) theoretical source signal (red line); (**b**) experimental source signal sent by the loudspeaker through the pipe (blue line) recorded during the controlled experiment.

**Figure 8:**
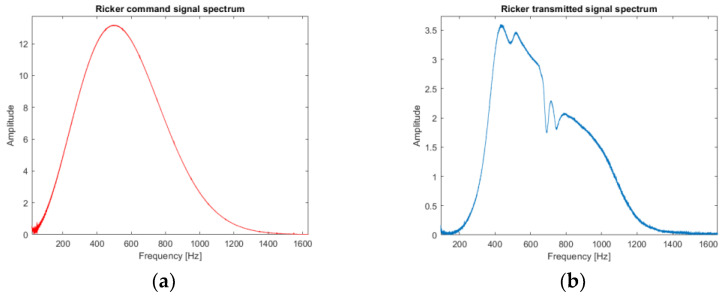
Fourier transform of the Ricker function: (**a**) theoretical Ricker spectrum (red line) showing the central frequency of the theoretical source around 500 Hz; (**b**) experimental Ricker spectrum (loudspeaker output; blue line) showing a central frequency around 500 Hz but distorted in relation to the command.

The first signal of interest is a Ricker function. The time response of the loudspeaker to a Ricker function control signal is presented in Figure 7 and the frequency response in Figure 8. The properties of this type of signal are used in the calculation of the propagator in the simulation of the propagation of the complete wave field by finite differences. The real measurements with a Ricker function emission are to compare the trend of the delay times with the simulations.

**Figure 9:**
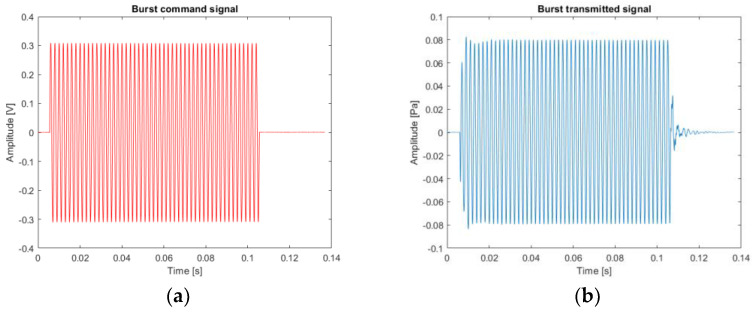
Example of the loudspeaker response (burst of 100 ms at 500 Hz): (**a**) theoretical source signal; (**b**) experimental source signal sent by the loudspeaker through the pipe.

**Figure 10:**
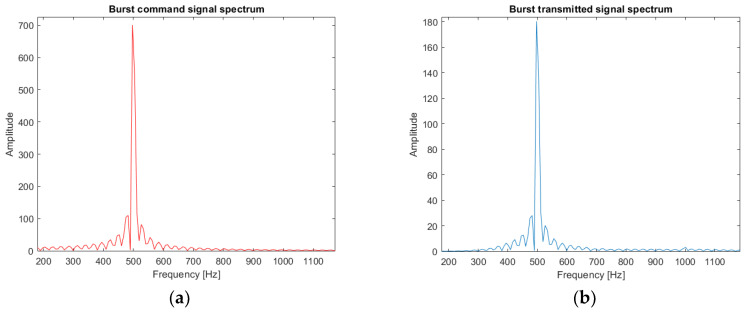
Fourier transform of the burst of 100 ms at 500 Hz: (**a**) theoretical burst spectrum; (**b**) experimental burst spectrum (loudspeaker output).

The second signal of interest is a monochromatic signal at 500 Hz, a burst signal. It is used to estimate the pipe depth. The time response of the loudspeaker to a burst control signal is presented in Figure 9 and the frequency response in Figure 10.

The loudspeaker distorts the burst less than the Ricker function, because the Ricker function is richer in frequency.

**Figure 11:**
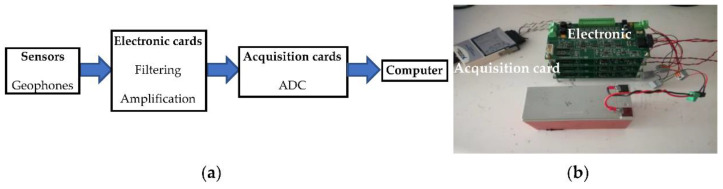
Experimental set-up of the reception chain: (**a**) scheme of the main components; (**b**) photograph of the material.

The acoustic reception chain is presented in Figure 11. The sensors are geophones that measure vibrations in their vertical axis. They are cylindrical with a diameter of 2.54 cm. During the measurements, a fatty substance was added between the sensors and the ground in order to obtain a better coupling. Geophones receive the signals that are filtered and amplified by electronic cards. At the input of electronic cards, signals are filtered using a high-pass filter with a cut-off frequency of 100 Hz, and the output, using a low-pass filter with a cut-off frequency of 100 kHz. This wide analog filtering allows us to digitally refine the filtering. The amplification is adjustable between 0 and 112 dB, which allows the amplification to be adjusted in the field. At the output of the electronic cards, the signals are digitized by the acquisition card; it is an analog-to-digital converter. The digitized signals are recovered on a computer.

**Figure 12:**
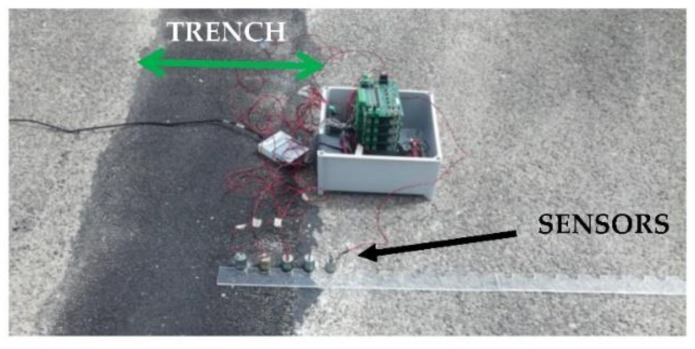
Example of positioning of the experimental device for signal acquisition (reception chain). The black arrow indicates the acquisition design composed of a line of five sensors and the support (graduated ruler), where the sensors are moved during the experiment. The green arrow indicates the trench where a pipe is buried. The box contains the electronic cards presented in Figure 11 and, on the left of the box, the acquisition card connected to the computer to digitalize the signals received from the buried pipe is shown.

Figure 12 shows an example of measurements taken on the semi-controlled test area. The trench and the sensors positioned perpendicular to the pipe passage can be observed. Sensors are spaced 5 cm apart.

#### 2.5.2. Experimental Results

Experimental measurements were performed on a semi-controlled test area. In this test area, the position of the pipe was known, and it was a polyethylene pipe, but the characteristics of the propagation medium were unknown. The propagation velocity is unknown.

To take the measurements, a line of five sensors was positioned perpendicular to the pipe route. To focus on the depth, it was assumed that the plumb of the pipe (S_X_) was known, and sensor 1 was placed just above the pipe. Sensors were spaced 0.2 m apart. The signal emitted in the pipe was a monochromatic signal at 500 Hz.

**Figure 13:**
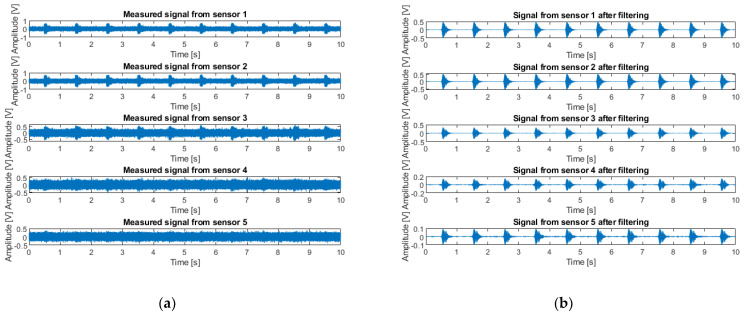
Example of signals measured by sensors (burst emission of 100 ms at 500 Hz): (**a**) signals received by sensors before digital filtering; (**b**) signals digitally filtered between 482 and 520 Hz.

In Figure 13, an example of signals measured by sensors is presented. The signal emitted by the loudspeaker is a burst of 100 ms at 500 Hz repeated every second. Figure 13a shows the signals received by the sensors before digital filtering, and Figure 13b shows the same signals digitally filtered between 480 and 520 Hz. Sensor 1 is the closest to the pipe and sensor 5 the farthest. The sensors farthest from the pipe receive the most attenuated signals.

Results on real signals are presented, for which depth estimation with the desired accuracy of 0.1 m (Table 2 and Table 3) is reached. For a depth of 0.42 m, an accuracy of 0.03 m (Table 2) is obtained. For a depth of 0.7 m, an accuracy of 0.05 (Table 3) is obtained.

**Figure 14:**
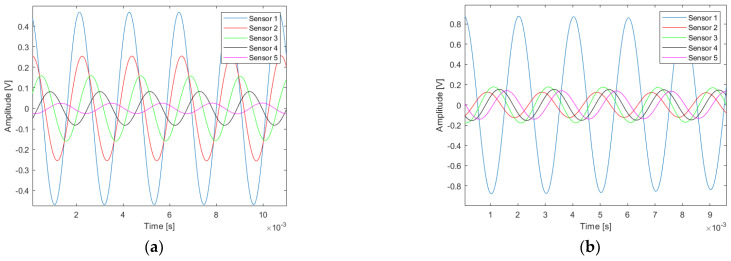
Example of real received signals digitally filtered between 480 and 520 Hz: (**a**) corresponding to Model M1; (**b**) not corresponding to Model M1.

Figure 14a shows measured signals (filtered between 480 and 520 Hz) on the test area that match with Model M1 (a zoom of Figure 13b). With Model M1, the more receivers are far from the source S, the more they should receive in a delayed way the information of the sound wave propagating in the pipe. It is on real signals of this type that the results, presented in Table 2 and Table 3, are obtained.

Figure 14b shows measured signals on the test area that do not match with Model M1. A phenomenon can be discerned several times, in that, sensors farthest from the source S receive the signal before the closest sensors. This phenomenon shows the role played by the vertical discontinuities (the trench) not included in Model M1.

It is needed to evolve the model to cover a greater number of situations. This is why an evolution of this model is presented in Section 3. Model M1 is sufficient when the transition is smooth or the medium weakly heterogeneous.

## 3. Modeling with Two Propagation Media (M2)

In this part, an evolution of the model is presented. Now sensors are placed inside and outside the trench. A vertically stratified change in medium is considered, which represents the trench in which the pipe is buried. First, the modeling of the propagation times is presented, and then the calculation of the intermediate variables is presented in more detail.

### 3.1. Propagation Time Modeling M2

#### 3.1.1. Presentation of the Propagation Time Model M2

**Figure 15:**
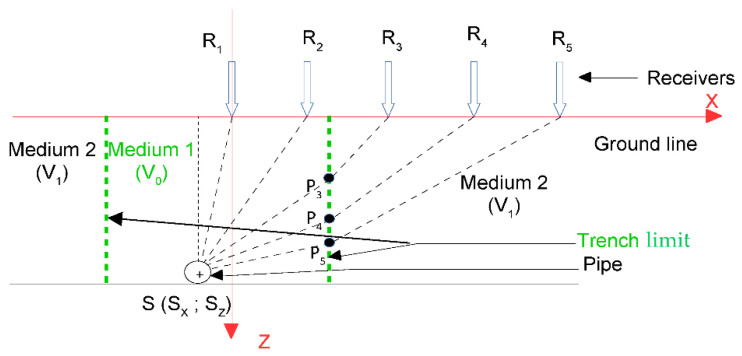
Scheme of the problem modeling in the case of two propagation media: Model M2.

In the problem modeling, a change in medium was added to take into account the trench (Figure 15). The pipe section was still represented using a point source S and the sensors R_i_ were arranged on the ground surface in the same way as before. An average velocity V_0_ was assigned to the propagation medium inside the trench and an average velocity V_1_ outside the trench. For the sensors outside the trench, new intermediate variables P_i_ appeared, which represented the interface points between the two media. For each R_i_ outside the trench, there was a corresponding P_i_.

We defined $\theta_{M2}$ as the vector of variables to be estimated in the case of Model M2.

(10) $$\theta_{M2}\;=\;{\left[\begin{array}{cccc} S_{X} & S_{Z} & V_{0} & V_{1} \end{array}\right]}^{T}$$

We noted $\tau_{i}\left(\theta_{M2}\right)$ as the wave travel time between the source S and the receiver R_i_ for Model M2. Two cases can be discerned:

- If R_i_ is inside the trench, it is returned to the single propagation medium case of Model M1 (Equation (2))
- If R_i_ is outside the trench, then

(11) $$\tau_{i}\left(\theta_{M2}\right)\;=\;\frac{\left|{S\;P}_{i}\right|}{V_{0}}\;+\;\frac{\left|P_{i}{\;R}_{i}\right|}{V_{1}}$$

In the following, only the case of R_i_ outside the trench is considered because the case R_i_ inside the trench was already covered in Section 2.

Relative delay times are also considered because the emission time of the source S is unknown. We note $\tau_{1i}\left(\theta_{M2}\right)$ as the relative delay time between the sensors R_1_ and R_i_ for Model M2.

(12) $$\tau_{1i}\left(\theta_{M2}\right)\;=\;\frac{\left|{S\;P}_{i}|\;-\;|{S\;R}_{1}\right|}{V_{0}}\;+\;\frac{\left|P_{i}{\;R}_{i}\right|}{V_{1}}$$

The modeling proposed in this paper depended on the interface point P_i_, which was unknown. We had to express P_i_ as a function of $\theta_{M2}$.

#### 3.1.2. Analytical Expression of the Interface Point P_i_

The variable $\tau_{1i}\left(\theta_{M2}\right)$ (Equation (12)) depended on the interface point P_i_ (P_iX_; P_iZ_). It was assumed that the X coordinate P_iX_ was known a priori (i.e., we knew the position of the medium change). However, we had no a priori information on the Z coordinate P_iZ_. We focused on the analytical expression of P_iZ_.

The Snell–Descartes law was considered at the interface between the two media. We can then write

(13) $$\frac{S_{Z}\;-\;P_{\mathrm{iZ}}}{\left|{S\;P}_{i}\right|}\;=\;\frac{P_{\mathrm{iZ}}}{\left|P_{i}{\;R}_{i}\right|}\frac{V_{0}}{V_{1}}$$

After squaring and developing, the following fourth-order equation was obtained:

(14) $$P_{\mathrm{iZ}}^{4}\;\left[V_{1}^{2}\;-\;V_{0}^{2}\right]\;\;+\;P_{\mathrm{iZ}}^{3}\;\left[-2R_{\mathrm{iZ}}V_{1}^{2}\;-\;2S_{Z}V_{1}^{2}\;+\;2S_{Z}V_{0}^{2}\right]\;\;+\;P_{\mathrm{iZ}}^{2}\;\left[S_{Z}^{2}V_{1}^{2}\;+\;R_{\mathrm{iZ}}^{2}V_{1}^{2}\;+\;V_{1}^{2}{\left(R_{\mathrm{iX}}\;-\;P_{\mathrm{iX}}\right)}^{2}\;+\;4R_{\mathrm{iZ}}S_{Z}V_{1}^{2}\;-\;S_{Z}^{2}V_{0}^{2}\;-\;V_{0}^{2}{\left(P_{\mathrm{iX}}\;-\;S_{X}\right)}^{2}\right]\;\;+\;P_{\mathrm{iZ}}\;\left[-2R_{\mathrm{iZ}}S_{Z}^{2}V_{1}^{2}\;-\;2S_{Z}R_{\mathrm{iZ}}^{2}V_{1}^{2}\;-\;2S_{Z}V_{1}^{2}{\left(R_{\mathrm{iX}}\;-\;P_{\mathrm{iX}}\right)}^{2}\right]\;\;+\;\left[R_{\mathrm{iZ}}^{2}S_{Z}^{2}V_{1}^{2}\;+\;S_{Z}^{2}V_{1}^{2}{\left(R_{\mathrm{iX}}\;-\;P_{\mathrm{iX}}\right)}^{2}\right]\;=\;0$$

We propose

(15) $$P_{\mathrm{iZ}}^{4}\;m_{1}\;+\;P_{\mathrm{iZ}}^{3}\;m_{2}\;+\;P_{\mathrm{iZ}}^{2}{\;m}_{3}\;+\;P_{\mathrm{iZ}}{\;m}_{4}\;+\;m_{5}\;=\;0$$

After solving Equation (15), we obtained

(16) $$P_{\mathrm{iZ}}\;=\;\frac{\sqrt{A}\;-\;\sqrt{A\;-\;2\left(g\;+\;A\;+\;\frac{u}{\sqrt{A}}\right)}}{2}\;-\;\frac{m_{2}}{4m_{1}}$$

with

(17) $$A\;=\;{\left(\frac{-K\;+\;\sqrt{K^{2}\;+\;\frac{4J^{3}}{27}}}{2}\right)}^{1/3}\;-\;\frac{J}{3{\left(\frac{-K\;+\;\sqrt{K^{2}\;+\;\frac{4J^{3}}{27}}}{2}\right)}^{1/3}}\;-\;\frac{2g}{3}\;,$$

(18) $$J\;=\;-\frac{g^{2}}{3}\;-\;4w\;,$$

(19) $$K\;=\;\frac{8\mathrm{wg}}{3}\;-\;\frac{2g^{3}}{27}\;-\;u^{2}\;,$$

(20) $$g\;=\;\frac{m_{3}}{m_{1}}\;-\;\frac{3m_{2}^{2}}{8m_{1}^{2}}\;,$$

(21) $$u\;=\;\frac{m_{4}}{m_{1}}\;-\;\frac{m_{2}m_{3}}{2m_{1}^{2}}\;+\;\frac{m_{2}^{3}}{8m_{1}^{3}}\;,$$

(22) $$w\;=\;\frac{m_{5}}{m_{1}}\;-\;\frac{m_{2}m_{4}}{4m_{1}^{2}}\;+\;\frac{m_{2}^{2}m_{3}}{16m_{1}^{3}}\;-\;\frac{3m_{2}^{4}}{256m_{1}^{4}}$$

$m_{1}$ to $m_{5}$ depended on $\theta_{M2}$ (Equation (10)), the position of sensors R_i_ (known) and the position of the medium change P_iX_ (known). The expression of the interface point depth P_iZ_ depended on $m_{i}$, so we expressed P_iZ_ as a function of variables of interest $\theta_{M2}$.

The theoretical delay times with Model M2 from $\theta_{M2}$ can be estimated following this approach.

### 3.2. Validation of Travel Time Estimate of M2 through Comparison with a Finite Difference Modeling of the Full Wavefield

Here, the travel times of Model M2 are compared with the full P-wavefield propagation modeling using finite differences in the same way as in Section 2.2. The simulations were performed for the same case study in order to compare the results.

The following set-up was considered: sensor 1 is the origin of the reference frame; the point source S is at a depth of S_Z_ = 0.7 m; sensor 1 is in line with the source S_X_ = 0 m; sensors are spatially distributed every 0. 05 m and move away from the source; the position of the change in medium is at P_X_ = 0.1 m; the average propagation velocity inside the trench is V_0_ = 300 m/s and outside the trench is V_1_ = 600 m/s. These values were proposed for the propagation velocities because they were close to those that would be estimated for the real data in the test site.

**Figure 16:**
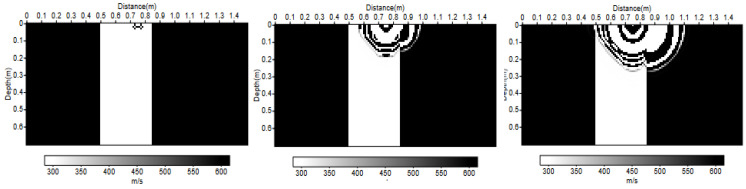
Physical velocity model and wavefront in the case of the full P-wavefield propagation modeling using the finite differences method.

To ensure simplicity in the simulation, the origin of the reference frame was translated, and the source was placed at the top and the sensors at the bottom, but they were symmetrical. In the simulation presented here, the source was placed at S (0.75; 0) and the sensors at Ri (0.75 + (i − 1)0.05; 0.7), but this corresponds well to the situation described above. In Figure 16, the physical model used for simulation and the wavefront propagation in the case of the full wavefield propagation modeling using finite differences are observed. Each panel is taken at a different time, and the evolution of the wavefront over time is observed. In this simulation, the source used was a Ricker function (Figure 7a and Figure 8a).

**Figure 17:**
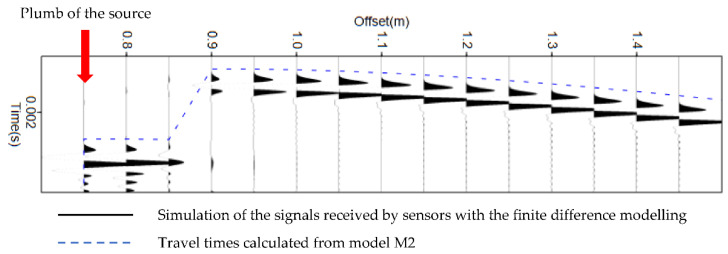
Seismograms obtained by full wavefield propagation using the finite differences method in the physical velocity model presented in Figure 16. The horizontal axis indicates the position of the sensors. The red arrow indicates the sensors located at the plumb of the source. The theoretical travel times calculated in the same model (M2) by the method proposed in Section 3.1 are plotted with a blue dashed line to assess the good agreement. The transmitted signal is considered as the same emitted Ricker function (Figure 7a).

The results of this simulation are presented in Figure 17. The abscissa represents the spatial position of the sensors, and the ordinate indicates the recorded time. The seismograms represent the simulation of the received signals using finite differences, and the travel time curve calculated from Model M2 is shown in blue. At the scale of the problem, Model M2 was in agreement with the more complex full wavefield model using finite differences. This allowed us to obtain a first validation of the travel time estimate of Model M2 through numerical simulation.

**Figure 18:**
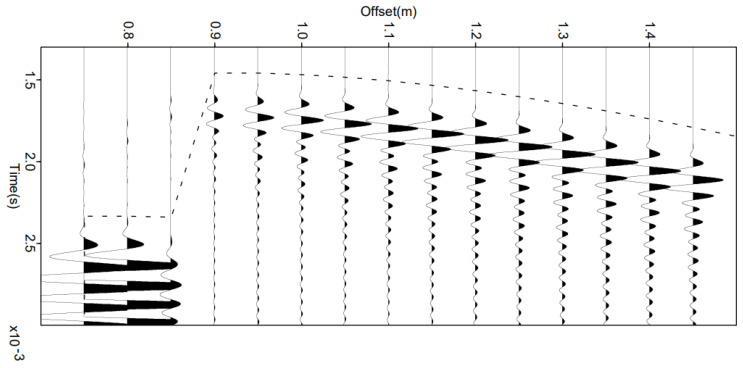
Seismograms obtained by full wavefield propagation using the finite differences method in the physical velocity model presented in Figure 16. The horizontal axis indicates the position of the sensors. The red arrow indicates the sensors located at the plumb of the source. The theoretical travel times calculated in the same model (M2) by the method proposed in Section 3.1 are plotted with a blue dashed line to assess the good agreement. We consider that the emitted signal was transformed by the loudspeaker (Figure 7b).

The same simulation is also carried out but using as source signal the signal actually transmitted by the loudspeaker (Figure 7b and Figure 8b). In Figure 18, a small shift between the travel times calculated with Model M2 and the simulations of the signals received using finite differences can be observed, but the trend of the curve remains correct.

### 3.3. Cramer–Rao Bound from Model M2

In this section, as in Section 2.3, the Cramer–Rao Bound (CRB) is calculated to obtain information on the estimation accuracy.

#### 3.3.1. Calculation of the Cramer–Rao Bound from Model M2

The CRB is calculated according to the Fisher information matrix, which is denoted by F.

(23) $$\mathrm{CRB}\left(\theta_{M2}\right)\;=\;F^{-1}\left(\theta_{M2}/\tau_{1i}\left(\theta_{M2}\right)\right)$$

where ./. is the known operator.

The Fisher information matrix is expressed

(24) $$F\left(\theta_{M2}/\tau_{1i}\left(\theta_{M2}\right)\right)\;=\;\overset{N}{\underset{i\;=\;2}{\sum }}\frac{1}{\mathrm{Var}\left(\tau_{1i}\left(\theta_{M2}\right)\right)}\nabla_{\theta_{M2}}\left(\tau_{1i}\left(\theta_{M2}\right)\right)\nabla_{\theta_{M2}}^{T}\left(\tau_{1i}\left(\theta_{M2}\right)\right)$$

where N is the number of sensors, $\mathrm{Var}\left(\tau_{1i}\left(\theta_{M2}\right)\right)$ is the variance of the relative delay time between sensor 1 and i, and $\nabla_{\theta_{M2}}\left[.\right]$ is the gradient operator as a function of $\theta_{M2}$.

The gradient of $\tau_{1i}\left(\theta_{M2}\right)$ is calculated from Equation (12),

(25) $$\nabla_{\theta_{M2}}\left(\tau_{1i}\left(\theta_{M2}\right)\right)\;=\;\nabla_{\theta_{M2}}\left(\frac{\left|{S\;P}_{i}\right|}{V_{0}}\right)\;-\;\nabla_{\theta_{M2}}\left(\frac{\left|{S\;R}_{1}\right|}{V_{0}}\right)\;+\;\nabla_{\theta_{M2}}\left(\frac{\left|P_{i}{\;R}_{i}\right|}{V_{1}}\right)$$

The calculation of the gradient can be decomposed as follows

(26) $$\nabla_{\theta_{M2}}\left(\frac{\left|{\mathrm{SP}}_{i}\right|}{V_{0}}\right)\;=\;{[\frac{\left(S_{X}\;-\;P_{\mathrm{iX}}\right)\;-\;\left(S_{Z}\;-\;P_{\mathrm{iZ}}\right)\;\frac{\partial }{\partial S_{X}}P_{\mathrm{iZ}}}{V_{0}|{\mathrm{SP}}_{i}|}\;\;\frac{\left(S_{Z}\;-\;P_{\mathrm{iZ}}\right)\left(1-\frac{\partial }{\partial S_{Z}}P_{\mathrm{iZ}}\right)}{V_{0}|{\mathrm{SP}}_{i}|}\;\;\frac{-V_{0}\left(S_{Z}\;-\;P_{\mathrm{iZ}}\right)\;\frac{\partial }{\partial V_{0}}P_{\mathrm{iZ}}\;-\;|{\mathrm{SP}}_{i}|{}^{2}}{V_{0}^{2}|{\mathrm{SP}}_{i}|}\;\;\frac{-\left(S_{Z}\;-\;P_{\mathrm{iZ}}\right)\;\frac{\partial }{\partial V_{1}}P_{\mathrm{iZ}}}{V_{0}|{\mathrm{SP}}_{i}|}]}^{T}$$

(27) $$\nabla_{\theta_{M2}}\left(\frac{\left|{\mathrm{SR}}_{1}\right|}{V_{0}}\right)\;=\;{\left[\frac{S_{X}\;-\;R_{1X}}{V_{0}|{S\;R}_{1}|}\;\;\frac{S_{Z}\;-\;R_{1Z}}{V_{0}|{S\;R}_{1}|}\;\;\frac{-|{S\;R}_{1}|}{V_{0}^{2}}\;\;0\right]}^{T}$$

(28) $$\nabla_{\theta_{M2}}\left(\frac{\left|P_{i}{\;R}_{i}\right|}{V_{1}}\right)\;=\;{\left[\frac{\left(P_{\mathrm{iZ}}\;-\;R_{\mathrm{iZ}}\right)\;\frac{\partial }{\partial S_{X}}P_{\mathrm{iZ}}}{V_{1}|P_{i}{\;R}_{i}|}\;\;\frac{\left(P_{\mathrm{iZ}}\;-\;R_{\mathrm{iZ}}\right)\;\frac{\partial }{\partial S_{Z}}P_{\mathrm{iZ}}}{V_{1}|P_{i\;}R_{i}|}\;\;\frac{\left(P_{\mathrm{iZ}}\;-\;R_{\mathrm{iZ}}\right)\;\frac{\partial }{\partial V_{0}}P_{\mathrm{iZ}}}{V_{1}|P_{i}{\;R}_{i}|}\;\;\frac{V_{1}\left(P_{\mathrm{iZ}}\;-\;R_{\mathrm{iZ}}\right)\;\frac{\partial }{\partial V_{1}}P_{\mathrm{iZ}}\;-\;|P_{i}{\;R}_{i}|{}^{2}}{V_{1}^{2}|P_{i}{\;R}_{i}|}\right]}^{T}$$

All these expressions depended on the gradient of P_iZ_. The gradient of P_iZ_, $\nabla_{\theta_{M2}}P_{\mathrm{iZ}}$, needed to be calculated to be able to calculate the CRB. This gradient calculation is presented in Appendix A to avoid overloading the text.

#### 3.3.2. Numerical Simulation Using the Cramer–Rao Bound from Model M2

The figures presented in this section are the result of numerical simulations. These results are not exhaustive and are used to give an idea of the impact of the variables of the problem on the accuracy of the depth estimation.

**Figure 19:**
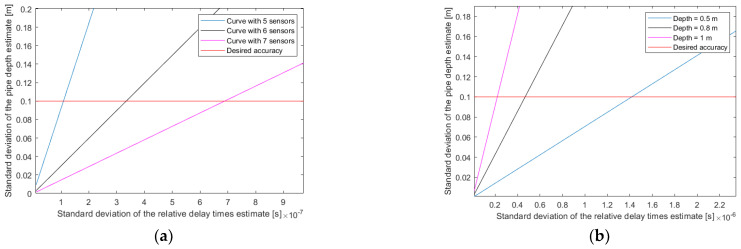
Depth error as a function of the error in the relative delay times: (**a**) for several numbers of sensors; (**b**) for several depths.

Figure 19 shows the evolution of the CRB on the depth as a function of the error in the relative delay times. For these simulations, the following situation is considered: the distance between sensors is 0.2 m; the plumb of the pipe (S_X_) is at 0 m; the average propagation velocity inside the trench (V_0_) is 300 m/s; the average propagation velocity outside the trench (V_1_) is 600 m/s; and the position of the change in medium (P_X_) is at 0.15 m.

For Figure 19a, a depth of 0.7 m is considered, and the curves for different numbers of sensors are observed. The accuracy constraint on the relative delay times is relaxed by increasing the number of sensors.

For Figure 19b, seven sensors are considered, and the curves for different depths are observed. The estimation of relative delay times should be more accurate as the depth increases. For example, here, with seven sensors, to obtain a precision of 0.1 m for the depth, the precision of the relative delay times is needed to be in a 0.1 μs range.

### 3.4. Validation of the Travel Time Model M2 on Real Data

To compare travel time modeling with real data, an experiment was performed on the semi-controlled test area in a case similar to the simulation in Section 3.2. The same signal as in the simulation, a Ricker, was emitted. Sensors were placed every 0.05 m perpendicular to the pipe passage (as in Figure 15). Since we did not have a large enough number of sensors, several successive measurements were performed by leaving one sensor fixed and moving the others to mesh the space and act as if there were a large number of sensors. The time bases were recalculated with respect to the fixed sensor of each measurement to act as if only one measurement had been realized with many sensors. This added an error in the travel times, but here, we were only interested in the trend of the evolution of the relative delay times in order to validate Model M2.

**Figure 20:**
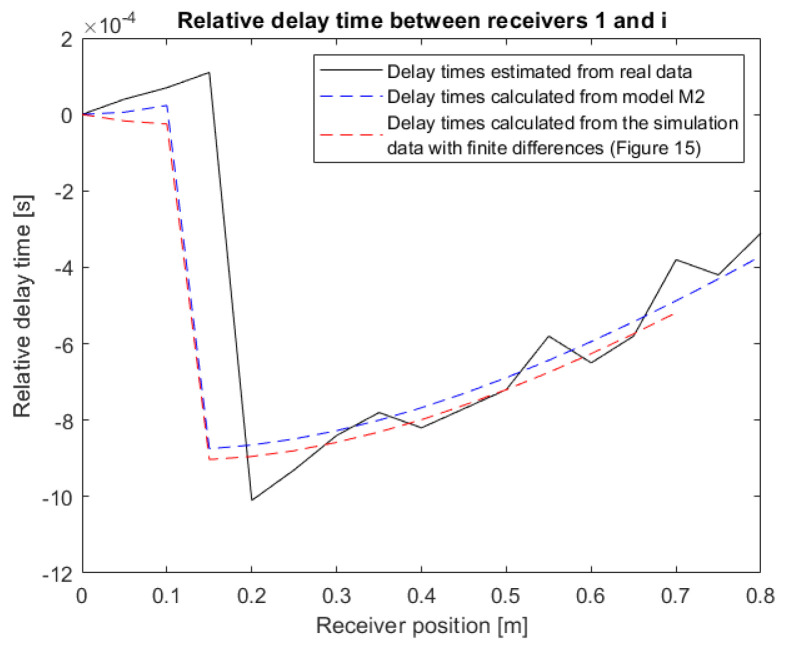
Comparison of the relative delay times between the real data and Model M2.

Sensor 1 was taken as a reference, which was the closest to the source S, to calculate the relative delay times. Figure 20 shows the evolution of the relative delay times between sensors 1 and i in black. These relative delay times were estimated using cross-correlation between the signal received by sensor 1 and that of sensor i.

The propagation velocities were unknown, a priori, for the semi-controlled test area. The velocities V_0_ and V_1_ of Model M2 can be estimated a posteriori by knowing the position of the source S in the test area. After determining all the variables in the test zone a posteriori, the relative delay times of Model M2 (blue curve of Figure 20) were calculated.

In the beginning, the relative delay times increased until the break that marked the change in environment. Then, they started to increase again. In the simulation (blue curve), the position of the change in medium was fixed at 0.1 m. On the real data (black curve), the change in medium, highlighted by the break of the curve, was instead between 0.15 and 0.2 m. The Model M2 is representative of reality. It is logical and is in agreement with the observation of real data.

### 3.5. Depth Estimation from Model M2

#### 3.5.1. Numerical Simulation with MUSIC Algorithm Adapted to Model M2

In this section, numerical simulations are performed to qualify the MUSIC estimator adapted to Model M2 (as in Section 2.4.2). The statistics of the estimator using the Monte Carlo method (1000 runs of noise) are presented. Consider the following situation 1: a depth (S_Z_) of 0.7 m, a propagation velocity (V_0_) of 300 m/s, a propagation velocity (V_1_) of 600 m/s, the plumb of the pipe (S_X_) at 0 m, and the position of the change in medium (P_X_) at 0.15 m. Seven sensors were spaced 0.2 m apart, the first one directly at the plumb of the pipe.

In Table 4, the statistics of the results of the estimator using the Monte Carlo method are presented. A total of 1000 runs of noise were performed. The theoretical propagation times between the source S and sensors were noised with a white Gaussian noise of standard deviation σ_noise_. The propagation times are in the millisecond range, and the propagation time differences with sensor 1 vary between 10^−5^ and 10^−3^ seconds. In this situation, an accuracy in a microsecond range is required on the delay times to obtain an accuracy of less than 0.1 m on the depth. These results confirm those presented in Section 3.3.2.

Now, the case with seventeen sensors spaced 0.05 m apart is presented. Consider the following situation 2: a depth (S_Z_) of 0.7 m, a propagation velocity (V_0_) of 300 m/s, a propagation velocity (V_1_) of 600 m/s, the plumb of the pipe (S_X_) at 0 m, and the position of the change in medium (P_X_) at 0.15 m. Seventeen sensors were spaced 0.05 m apart, the first one directly at the plumb of the pipe.

The results in Table 5 show that an error of the order of microseconds in delay times allows an accuracy of 0.1 m on the depth.

#### 3.5.2. Estimation on Real Data

At least seven sensors are required (Section 3.3.2) to use the MUSIC algorithm adapted to Model M2. As mentioned in Section 3.4, we only had five sensors. The sensors were moved to perform successive measurements. The different measurements are then not synchronized. Therefore, our estimator, based on MUSIC, cannot be used on these measurements.

However, results evaluated from the delay times between the sensors are presented in Figure 20 (black curve). These delay times were obtained after retiming, introducing additional errors.

The criterion presented here is the inverse of the squared error between the estimated delay times and the delay times calculated by Model M2.

The values of the criterion are obtained by varying the variables to be estimated: the depth (S_Z_), the velocity inside the trench (V_0_) and the velocity outside the trench (V_1_). The lateral position of the pipe (S_X_) and the position of the medium change (P_X_) are considered known. Indeed, S_X_ estimated with the GasTracker tool and P_X_ from the temporal break are observed in Figure 20.

**Figure 21:**
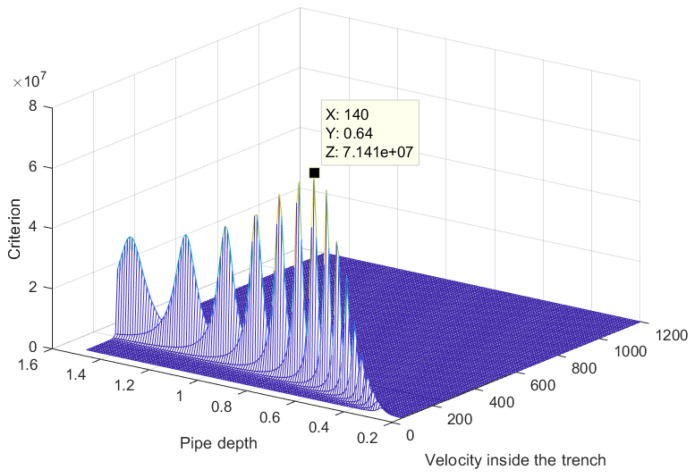
The inverse of the squared error between the estimated delay times and the delay times calculated by Model M2. For this display, the velocity outside the trench V1 is fixed at the estimated value.

Figure 21 is a display of the obtained criterion. The estimation results are presented in Table 6. The error in the depth is less than 0.1 m. In addition, the velocity estimates are really small, which could be due to the large error in the delay times. The parameters S_X_ and P_X_ are fixed by an a priori estimate. It would be interesting to also vary these parameters around their estimated position.

These results obtained with least squares are encouraging, but we would like to have a more discriminating criterion. The next step of our work will be to test the MUSIC algorithm on real synchronous data in order to have a criterion with a better resolution power.

## 4. Conclusions and Future Perspectives

We developed a method using several sensors positioned perpendicular to the passage of the pipe. This allows us to model the problem in a plane orthogonal to the passage of the pipe. The vibrating section of the pipe is represented by a point source.

In the first section, an initial model of the problem was defined by considering a single propagation medium (M1), i.e., all sensors are located at the ground surface inside the trench. This Model M1 was validated, on the scale of the problem, by comparing it to a more complete modeling (full wavefield modeling by finite differences). The Cramer–Rao bound was calculated to obtain theoretical information on the accuracy reached by the variables, in particular on the relative delay times. The error in the positioning of the sensors (e.g., line of sensors not perpendicular to the pipe) induces an error in the travel times. This is why, in these simulations and theoretical studies, the error in the depth estimation was quantified as a function of the error in the delay times. The MUSIC algorithm was adapted to Model M1, and then the algorithm was tested on real data. This allowed us to question the model and thus, to advance it. The results obtained from the M1 model show that the depth estimates reach the desired accuracy of 0.1m.

In the second section, a second model of the problem was defined by considering two vertically stratified propagation media (M2), i.e., sensors are located at the ground surface inside and outside the trench, according to the information obtained from experiments onto a test area. This model was validated in two steps, first through numerical simulation by comparing it to a more complete model, then by comparing it to real data. The Cramer–Rao bound was also calculated to obtain theoretical information on the accuracy reached by the variables. The MUSIC algorithm was adapted to this Model M2 and was tested in simulation.

Model M2 aims to cover the case of a marked change in medium (inside/outside the trench). The results obtained with the Cramér–Rao bound show that seven or eight sensors would be needed to obtain a satisfactory depth estimate. Not having real synchronous measurements realized with eight sensors, a least squares criterion between the delay times estimated from the real signals and those given by Model M2 was established. The results obtained on real data reach the desired accuracy.

In our future work, with more sensors, we will test the estimator based on the MUSIC algorithm adapted to Model M2 on real data. Depending on the feedback from these experiments, we will be able to continue to evolve the model by potentially considering other propagation media, such as the layer of sand surrounding the pipe or the layer of tar covering the ground. This aims to produce a model closer to real situations that provide the fastest possible result to be able to apply the method in the field in real-time.

## Figures and Tables

**Table 1:** Statistics of the MUSIC estimator adapted to Model M1 using the Monte Carlo method (for 1000 runs, 5 sensors spaced 0.2 apart). Noise is applied to the propagation times, which are in the order of a millisecond.

		Mean	Standard Deviation	True Value
Numerical Simulation 1 with 5 sensors and σ_noise_ = 1 × 10^−7^	Depth S_Z_ (m)	0.7056	0.0486	0.7
	Average velocity V_0_ (m/s)	494	17	500
	Plumb of the pipe S_X_ (m)	0.0047	0.0065	0
Numerical Simulation 2 with 6 sensors and σ_noise_ = 5 × 10^−7^	Depth S_Z_ (m)	0.6658	0.0317	0.7
	Average velocity V_0_ (m/s)	499	7	500
	Plumb of the pipe S_X_ (m)	0.0172	0.0109	0
Numerical Simulation 3 with 7 sensors and σ_noise_ = 1 × 10^−6^	Depth S_Z_ (m)	0.7386	0.0466	0.7
	Average velocity V_0_ (m/s)	501	9	500
	Plumb of the pipe S_X_ (m)	0.0325	0.0315	0

**Table 2:** First example of estimation obtained with the MUSIC algorithm adapted to the problem and to Model M1 (5 sensors spaced 0.2 m apart).

	Estimate Value	Reference Value
Depth (S_Z_) (m)	0.39	0.42
Average velocity (V_0_) (m/s)	360	unknown

**Table 3:** Second example of estimation obtained with the MUSIC algorithm adapted to the problem and to Model M1 (5 sensors spaced 0.2 m apart).

	Estimate Value	Reference Value
Depth (S_Z_) (m)	0.75	0.70
Average velocity (V_0_) (m/s)	540	unknown

**Table 4:** Statistics of the MUSIC estimator adapted to Model M2 using the Monte Carlo method in situation 1 (7 sensors spaced 0.2 m apart). Noise is applied to the propagation times, which are in the order of a millisecond.

		Mean	Standard Deviation	True Value
Numerical Simulation 1 with σ_noise_ = 5 × 10^−6^	Depth S_Z_ (m)	0.7294	0.0849	0.7
	Average velocity V_0_ (m/s)	293	19	300
	Average velocity V_1_ (m/s)	592	41	600
Numerical Simulation 2 with σ_noise_ = 5 × 10^−5^	Depth S_Z_ (m)	0.6666	0.2246	0.7
	Average velocity V_0_ (m/s)	261	58	300
	Average velocity V_1_ (m/s)	638	128	600

**Table 5:** Statistics of the MUSIC estimator adapted to Model M2 using the Monte Carlo method in situation 2 (17 sensors spaced 0.05 m apart). Noise is applied to the propagation times, which are in the order of a millisecond.

		Mean	Standard Deviation	True Value
Numerical Simulation 1 with σ_noise_ = 5 × 10^−6^	Depth S_Z_ (m)	0.6604	0.0704	0.7
	Average velocity V_0_ (m/s)	293	18	300
	Average velocity V_1_ (m/s)	611	79	600

**Table 6:** Estimation obtained with least squares from Model M2.

	Estimate Value	Reference Value
Depth (S_Z_) (m)	0.64	0.70
Average velocity inside the trench (V_0_) (m/s)	140	unknown
Average velocity outside the trench (V_1_) (m/s)	250	unknown

## Data Availability

Not applicable.

## Appendix A

### Calculation of the Gradient of P_iZ_

In this section, the gradient of P_iZ_ is expressed with respect to $\theta_{M2}$. The notations from Section 3.1.2 are used in the calculation of P_iZ_.

From Equation (16),

(A1) $$\nabla_{\theta_{M2}}P_{\mathrm{iZ}}\;=\;\frac{1}{2}\left(\frac{1}{2\sqrt{A}}\;\nabla_{\theta_{M2}}\left(A\right)\;-\;\frac{\nabla_{\theta_{M2}}\left(A\right)\;-\;2\left[\nabla_{\theta_{M2}}\left(g\right)\;+\;\nabla_{\theta_{M2}}\left(A\right)\;+\;\nabla_{\theta_{M2}}\left(\frac{u}{\sqrt{A}}\right)\right]}{2\sqrt{A\;-\;2\left(g\;+\;A\;+\;\frac{u}{\sqrt{A}}\right)}}\right)\;-\;\frac{m_{1}\nabla_{\theta_{M2}}\left(m_{2}\right)\;-\;m_{2}\nabla_{\theta_{M2}}\left(m_{1}\right)}{4{\;m}_{1}^{2}}$$

The gradients of all intermediate variables are calculated (A, J, K, g, u, w, m_1_, m_2_, m_3_, m_4_, m_5_) to express $\nabla_{\theta_{M2}}P_{\mathrm{iZ}}$. The variable A (Equation (17)) is decomposed into three terms to ensure that the calculation is still readable.

(A2) $$A\;=\;A_{1}\;+\;A_{2}\;+\;A_{3}$$

with

(A3) $$A_{1}\;=\;{\left(\frac{-K\;+\;\sqrt{K^{2}\;+\;\frac{4J^{3}}{27}}}{2}\right)}^{1/3},A_{2}\;=\;-\frac{J}{3{\left(\frac{-K\;+\;\sqrt{K^{2}\;+\;\frac{4J^{3}}{27}}}{2}\right)}^{1/3}}\;,A_{3}\;=\;-\frac{2g}{3}$$

The gradient of each term is calculated.

(A4) $$\nabla_{\theta_{M2}}\left(A_{1}\right)\;=\;\frac{-\nabla_{\theta_{M2}}\left(K\right)\;+\;\frac{2K\nabla_{\theta_{M2}}\left(K\right)\;+\;\frac{12J^{2}}{27}\nabla_{\theta_{M2}}\left(J\right)}{2\sqrt{K^{2}\;+\;\frac{4J^{3}}{27}}}}{6{\left(\frac{-K\;+\;\sqrt{K^{2}\;+\;\frac{4J^{3}}{27}}}{2}\right)}^{2/3}}$$

(A5) $$\nabla_{\theta_{M2}}\left(A_{2}\right)\;=\;-\frac{1}{3}\frac{\nabla_{\theta_{M2}}\left(J\right){\left(\frac{-K\;+\;\sqrt{K^{2}\;+\;\frac{4J^{3}}{27}}}{2}\right)}^{1/3}\;-\;J\nabla_{\theta_{M2}}\left(A_{1}\right)}{{\left(\frac{\;-\;K\;+\;\sqrt{K^{2}\;+\;\frac{4J^{3}}{27}}}{2}\right)}^{2/3}}$$

(A6) $$\nabla_{\theta_{M2}}\left(A_{3}\right)\;=\;-\frac{2}{3}\nabla_{\theta_{M2}}\left(g\right)$$

Once the gradient of A was calculated, the gradients of J and K can be calculated (Equations (18) and (19)).

(A7) $$\nabla_{\theta_{M2}}\left(J\right)\;=\;-\frac{2g}{3}\nabla_{\theta_{M2}}\left(g\right)\;-\;4\nabla_{\theta_{M2}}\left(w\right)$$

(A8) $$\nabla_{\theta_{M2}}\left(K\right)\;=\;\frac{8}{3}\left[g\;\nabla_{\theta_{M2}}\left(w\right)\;+\;w\;\nabla_{\theta_{M2}}\left(g\right)\right]\;-\;\frac{2g^{2}}{9}\nabla_{\theta_{M2}}\left(g\right)\;-\;2u\nabla_{\theta_{M2}}\left(u\right)$$

The calculation of the gradients of g, u and w can be realized (Equations (20)–(22)).

(A9) $$\nabla_{\theta_{M2}}\left(g\right)\;=\;\frac{m_{1}\nabla_{\theta_{M2}}\left(m_{3}\right)\;-\;m_{3}\nabla_{\theta_{M2}}\left(m_{1}\right)}{m_{1}^{2}}\;-\;\frac{3}{4}\frac{m_{2}m_{1}\nabla_{\theta_{M2}}\left(m_{2}\right)\;-\;m_{2}^{2}\nabla_{\theta_{M2}}\left(m_{1}\right)}{m_{1}^{3}}$$

(A10) $$\nabla_{\theta_{M2}}\left(u\right)\;=\;\frac{m_{1}\nabla_{\theta_{M2}}\left(m_{4}\right)\;-\;m_{4}\nabla_{\theta_{M2}}\left(m_{1}\right)}{m_{1}^{2}}\;-\;\frac{1}{2}\frac{\left[m_{2}\nabla_{\theta_{M2}}\left(m_{3}\right)\;+\;m_{3}\nabla_{\theta_{M2}}\left(m_{2}\right)\right]m_{1}\;-\;2m_{3}m_{2}\nabla_{\theta_{M2}}\left(m_{1}\right)}{m_{1}^{3}}\;+\;\frac{3}{8}\frac{m_{1}m_{2}^{2}\;\nabla_{\theta_{M2}}\left(m_{2}\right)\;-\;m_{2}^{3}\;\nabla_{\theta_{M2}}\left(m_{1}\right)}{m_{1}^{4}}$$

(A11) $$\nabla_{\theta_{M2}}\left(w\right)\;=\;\{\begin{array}{cc} & \frac{m_{1}\;\nabla_{\theta_{M2}}\left(m_{5}\right)\;-\;m_{5}\;\nabla_{\theta_{M2}}\left(m_{1}\right)}{m_{1}^{2}}\;-\;\frac{1}{4}\frac{\left[m_{2}\;\nabla_{\theta_{M2}}\left(m_{4}\right)\;+\;m_{4}\;\nabla_{\theta_{M2}}\left(m_{2}\right)\right]m_{1}\;-\;2m_{2}m_{4}\;\nabla_{\theta_{M2}}\left(m_{1}\right)}{m_{1}^{3}}\\ \;+\;\frac{1}{16} & \frac{\left[m_{2}^{2}\;\nabla_{\theta_{M2}}\left(m_{3}\right)\;+\;2m_{3}m_{2}\;\nabla_{\theta_{M2}}\left(m_{2}\right)\right]m_{1}\;-\;3m_{3}m_{2}^{2}\;\nabla_{\theta_{M2}}\left(m_{1}\right)}{m_{1}^{4}}\;-\;\frac{3}{64}\frac{m_{1}m_{2}^{3}\;\nabla_{\theta_{M2}}\left(m_{2}\right)\;-\;m_{2}^{4}\;\nabla_{\theta_{M2}}\left(m_{1}\right)}{m_{1}^{5}} \end{array}$$

Then, the gradients from m_1_ to m_5_, on which all other gradients depended, were calculated.

(A12) $$\nabla_{\theta_{M2}}\left(m_{1}\right)\;=\;{\left[\begin{array}{ccccccc} 0 & & 0 & & \;-\;2V_{0} & & 2V_{1} \end{array}\right]}^{T}$$

(A13) $$\nabla_{\theta_{M2}}m_{2}\;=\;{\left[\begin{array}{ccccccc} 0 & & 2[V_{0}^{2}\;-\;V_{1}^{2}] & & 4S_{Z}V_{0} & & -4V_{1}[R_{\mathrm{iZ}}\;+\;S_{Z}] \end{array}\right]}^{T}$$

(A14) $$\nabla_{\theta_{M2}}\left(m_{3}\right)\;=\;{\left[\begin{array}{ccccccc} 2V_{0}^{2}\left(P_{\mathrm{iX}}\;-\;S_{X}\right) & & 2S_{Z}V_{1}^{2}\;+\;4R_{\mathrm{iZ}}V_{1}^{2}\;-\;2S_{Z}V_{0}^{2} & & -2V_{0}\left[S_{Z}^{2}\;+\;{\left(P_{\mathrm{iX}}\;-\;S_{X}\right)}^{2}\right] & & 2V_{1}\left[S_{Z}^{2}\;+\;R_{\mathrm{iZ}}^{2}\;+\;{\left(R_{\mathrm{iX}}\;-\;P_{\mathrm{iX}}\right)}^{2}\;+\;4R_{\mathrm{iZ}}S_{Z}\right] \end{array}\right]}^{T}$$

(A15) $$\nabla_{\theta_{M2}}\left(m_{4}\right)\;=\;{\left[\begin{array}{ccccccc} 0 & & -2V_{1}^{2}\left[2R_{\mathrm{iZ}}S_{Z}\;+\;R_{\mathrm{iZ}}^{2}\;+\;{\left(R_{\mathrm{iX}}\;-\;P_{\mathrm{iX}}\right)}^{2}\right] & & 0 & & -4V_{1}S_{Z}\left[R_{\mathrm{iZ}}S_{Z}\;+\;R_{\mathrm{iZ}}^{2}\;+\;{\left(R_{\mathrm{iX}}\;-\;P_{\mathrm{iX}}\right)}^{2}\right] \end{array}\right]}^{T}$$

(A16) $$\nabla_{\theta_{M2}}\left(m_{5}\right)\;=\;{\left[\begin{array}{ccccccc} 0 & & 2S_{Z}V_{1}^{2}\left[R_{\mathrm{iZ}}^{2}\;+\;{\left(R_{\mathrm{iX}}\;-\;P_{\mathrm{iX}}\right)}^{2}\right] & & 0 & & 2S_{Z}^{2}V_{1}\left[R_{\mathrm{iZ}}^{2}\;+\;{\left(R_{\mathrm{iX}}\;-\;P_{\mathrm{iX}}\right)}^{2}\right] \end{array}\right]}^{T}$$

The gradients of m_1_ to m_5_ were calculated and expressed according to the gradients of P_iZ_.
